# From Hop to Beer: Influence of Different Organic Foliar Fertilisation Treatments on Hop Oil Profile and Derived Beers’ Flavour

**DOI:** 10.3390/plants12091861

**Published:** 2023-04-30

**Authors:** Margherita Rodolfi, Antonio Valentoni, Luca Pretti, Manuela Sanna, Simone Guidotti, Ilaria Marchioni, Tommaso Ganino

**Affiliations:** 1 Department of Food and Drug, University of Parma, 43124 Parma, Italy; 2 Porto Conte Ricerche S.r.l., 07041 Alghero, Italy; 3 Lab Service Analytica S.r.l., Via Emilia, 51/c, 40011 Anzola dell’Emilia, Italy; 4 National Research Council, Institute of BioEconomy (IBE), Via Madonna del Piano, 10, 50019 Sesto Fiorentino, Italy

**Keywords:** hop nutrition, beer flavour, hop aroma, sensory evaluation, electronic nose, GC-MS, HS-SPME-GC-MS

## Abstract

Foliar fertilisation is known to influence the physiological response of *Humulus lupulus* (hop plants), but its effect on the flavour profile of beer still has to be investigated. By comparing the effects of four fertilisation treatments, this study aims at determining whether different foliar fertilisation treatments have a significant impact on hop plants’ aromatic quality and that of the beer produced. Hop cones harvested from each experimental treatment were brewed to obtain five single dry-hopped beers, which were subsequently analysed. Gas chromatography–mass spectrometry (GC-MS) and electronic nose (Cyranose 320) analyses were performed on the hop cones, while headspace solid-phase microextraction–gas chromatography–mass spectrometry HS-SPME-GC-MS, electronic nose and sensory analyses were carried out on the beers produced. The analyses not only allowed for a differentiation between the hops from the four fertilisation treatments and the control but also enabled a differentiation between the beers produced for their identification. Sensory evaluation revealed consumer preferences regarding the dry-hopped beers analysed, evidencing their distinctive features, including significant differences in both aroma and flavour.

## 1. Introduction

Hops (*Humulus lupulus* L.) are the most expensive ingredient for breweries, used for their aromatic, preservative and bittering properties. Hop cultivation requires a large amount of nutrient input. In particular, as this species exhibits rapid growth in spring (it can grow up to 25 cm per day), the utilisation of high amounts of nutrients, especially nitrogen, is needed [1]. The use of different types of nutrients can influence both the yield and quality of plants [2,3,4] and, therefore, the quality of the processed product. In grape cultivation, fertilisation is part of the *terroir* effect, since it can affect wine quality not only by modifying grape microflora but also by affecting secondary metabolism [3,5]. Webster et al. [5] observed an effect of nitrogen (N) fertilisation on grape production, which highlighted significant differences in the monoterpene composition of wines and their sensorial features. Similar findings have also been reported for the tea tree (*Camelia sinensis* L.): several studies have shown that foliar fertilisation affects the aromatic compounds of leaves and teas. The research by Liu et al. [6], in particular, found a correlation between N application and the increment in (E)-2-hexenal and (Z)-3-hexanol, both of which are responsible for the typical greenish odour of green tea. Terpene, monoterpenoid and sesquiterpenoid biosynthesis also increased in *Chrysanthemum Boreale* (Makino) flowers following NPK (nitrogen, phosphorus and potassium) and lime fertilisation treatments [7].

In this context, the hop secondary metabolism is known to be influenced by different factors, such as the terroir [8,9,10], biotic and abiotic stress [11,12], and plant nutrition [10,13,14]. Traditionally, the most commonly used N fertilisers include ureas, coated ureas, ammonium nitrate, ammonium sulfate (highly acidifying), calcium nitrate and potassium nitrate [15]. Other synthesis fertilisers rich in N, P and K are commonly used for hop nutrition during the growing season [16]. Over recent decades, the agricultural world, including the brewery sector, has shown particular interest in organic production and manuring, as a result of an increased human awareness of environmental issues, which has led, in turn, to new research into organic raw materials [17,18]. This can be found in hop cultivation, where there is an increase in organic farming methods. For example, differences in hop cone quality have been found in an experiment comparing hops cultivated organically (probiotic microorganisms and plant extracts, horse manure and probiotic microorganisms) with those undergoing conventional manuring [19]. Organic hops exhibited a higher content not only of xanthohumol, proanthocyanidine and flavan-3-ol but also of alpha acids when compared with cones cultivated using conventional manuring. The impact of agronomic practices on hop quality is also evidenced in a study on different hop cultivars by Kishimoto et al. [10], where hops cultivated using copper sulphate exhibited a quantitative decrease in the aromatic profile of 4-mercapto-4-methylpentan-2-one, characterized by a blackcurrant-like note.

In beer production, the aromatic fraction contained in the essential oil of hops gives the beer a unique character. Therefore, the use of different hop fertilisation methods, acting directly or indirectly in secondary metabolism and terpene biosynthesis, could affect beer quality and sensory characteristics [20]. Similar sensory differences have previously been observed in a study on consumer preferences [21], where beers obtained using different fertilised hops were given to consumers, who effectively perceived differences in beer quality. This result highlights the importance of hop fertilisation [21]. Moreover, considering the increased interest in sustainability, the identification of a fertilisation method that could meet the needs of hop producers, brewers and the environment becomes more urgent.

At present, only fragmented data are available in the literature in regards to hop and beer quality and foliar fertilisation treatments. No studies have been performed including both these aspects in the same experimental design. In the present research, for the first time, the effect of different organic foliar fertilisation regimes on hops’ essential oil and beers was investigated, using a multidisciplinary approach for their characterisation, i.e., GC, Cyranose 320 and panel analysis.

## 2. Results and Discussion

### 2.1. Fertilisation Experiment

During the growing season, four organic foliar fertiliser mixtures, based principally on marine algae, amino acids and microelements (Treatment 1, Treatment 2, Treatment 3 and Treatment 4, namely T1, T2, T3 and T4) (treatment detail in Table S1), were tested on the same cultivar (Cascade) compared to a control (C) with no foliar fertilisation. The nutrient mixture was applied as foliar treatment. The hop yard has a loamy clay soil, with a pH of 7.33, Electric Conductivity (EC) of 264 µS/cm and Cationic Exchange Capacity (CEC) of 30.86 meq/100 g (other physical and chemical properties of the soil are reported in the Material and Methods section, Table 6). The experimental design and the first results of the experiment regarding physiological measurements have already been published [22]. The amounts of macro- and microelements applied to the hop plants are indicated in Table 1 and the fertiliser mixtures are indicated in Table S1. Non-treated plants were also considered, representing the control. For each treatment, three independent replicates were used, with each replicate consisting of 150 plants. Each treatment was separated from the next by five rows of untreated plants.

### 2.2. Plant Material Characterisation

Hop cone bitter acids and essential oils have been previously quantified by Rodolfi et al. [22] using methanol extraction and HPLC-UV analysis and steam distillation with a Clevenger apparatus for 4 h.

Bitter acids and essential oil yields obtained previously together with the moisture percentage per hop treatment are summarised in Table 2 in order to give a description of the starting material. The moisture content (g/100 g) of hop cone samples was evaluated by means of gravimetric analysis following the standard method [23].

### 2.3. Hop Cone Analysis

#### 2.3.1. GC-MS Hop Oil Analysis

In hops, the essential oil composition is influenced by several biotic and abiotic factors, plant nutrition being one of them. Fertilisation treatments could either be a useful tool to obtain optimum crop yields and quality results or, conversely, could be a source of stress for plants. From the GC-MS analysis of the cones coming from the 5 different fertilisation treatments (Figure S1), 16 terpenes were identified (Table 3), corresponding to 89 ± 1% of the total peak area. From these results, it was possible to observe differences in the aromatic profile: eight compounds showed statistical differences within the studied samples (Table 3), namely β-pinene, β-myrcene, β-caryophyllne, α-humulene, muurolene, β-selinene, α-selinene and τ-cadinene. The data in the table show a statistical difference in general between the treatments and the control, whereas there are slight significant differences between the treatments in essential oil composition (muurolene). The data demonstrate that the essential oils composition is less influenced by the different mixture of foliar fertilisation applied if compared to the resin composition (Table 2) [22]. The results demonstrate the complicated correlation between the nutrient input and plant response.

The compounds β-pinene and β-myrcene were less present in the C (control samples) condition, highlighting the impact of fertilisation treatments on aromatic compound biosynthesis. In contrast, the majority of the sesquiterpenes were found in higher amounts in the C (Table 3). These results are to some extent in line with those reported in a study on *Achyrocline satureioides* (Lam.) DC., where plants subjected to different nitrogen levels and plant spacing exhibited significant differences in α-humulene, β-caryophyllene and α-pinene content [26]. This study also highlighted a greater amount of α-pinene when plants were treated with either 60 or 120 t/ha of nitrogen and with 100 × 100 cm spacing, while amounts of α-humulene and β-caryophyllene were higher with a supply of 60 t/ha of N and 50 × 50 cm spacing. Similarly, in our study the presence of β-pinene and β-myrcene was higher in T1, T2, T3 and T4, where there was a greater quantity of nitrogen added. In contrast, the presence of β-caryophyllne, α-humulene, muurolene, β-selinene, α-selinene and τ-cadinene was higher in C (no N supply) [26]. Similar results have been found for sage (*Salvia officinalis* L.) subjected to different nitrogen levels, where α-humulene percentages were higher in samples with no nitrogen added [27].

The compounds β-pinene and β-myrcene (monoterpenes) together with β-caryophyllne, α-humulene, muurolene, β-selinene, α-selinene and τ-cadinene (sesquiterpenes) result from two different metabolic pathways: monoterpenes are synthesised by the mevalonate (MVA) pathway in the cytoplasm, whereas sesquiterpenes derive from the 2-C-methyl-D-erythritol 4-phosphate (MEP) pathway in plastids. According to Vilanova et al. [28] fertilisation could favour one pathway while hampering the other.

In a study on aromatic plants, Ormeño et al. [29] did not observe compost supply effects on the *Rosmarinus officinalis* terpene concentration in glandular trichomes, even if an increase in biomass production was detected. This might suggest that applied treatments do not contribute to the development of glandular trichomes to such a large degree in Mediterranean plants, since they need to avoid related energy costs as a plant maintenance strategy in arid periods. However, this finding is not in line with our study, where an increase in monoterpenes and a decrease in monoterpenes were observed. The level of sesquiterpenes in our samples, in particular, seems to have been reduced by high levels of N. A possible explanation, which is in accordance with the “excess carbon hypothesis”, could be that carbon-based secondary compounds decrease as a result of increased carbon allocation to growth in response to high nutrient availability [30]. In a study on *Phoebe bournei* (Hemsley) [31], a reduction in the sesquiterpene content was observed when compost was used (compared to treatments using NPK and no fertiliser), with the alteration in sesquiterpene biosynthesis being related to the up- or downregulation of the genes involved in the promotion or inhibition of important enzymes.

#### 2.3.2. Cyranose 320 Analysis on Hop Cones

**Figure 1:**
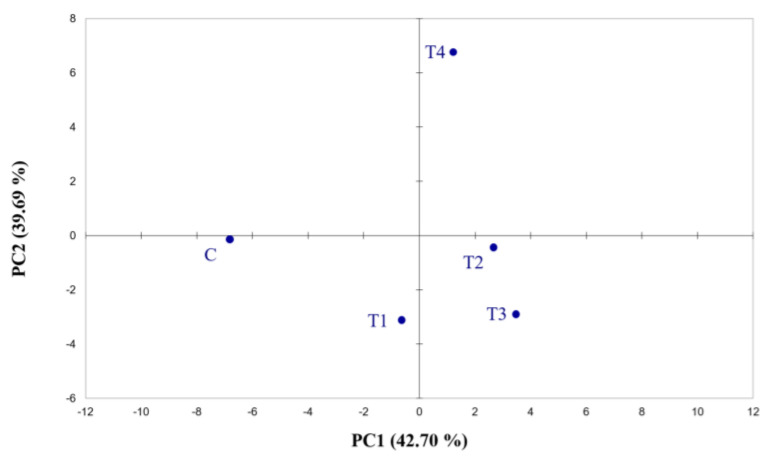
Score plots obtained from the PCA of Cyranose 320 sensor signals on hop cones across the fertilisation treatments. The analysis was performed using Pearson’s method (*p* ≤ 0.05). C = control treatment; T1 = Treatment 1; T2 = Treatment 2; T3 = Treatment 3; T4 = Treatment 4.

Electronic nose output demonstrates the ability of the instrument to differentiate among the samples belonging to different fertilisation treatments. The data collected by the 32 e-nose sensors were analysed using principal component analysis (Figure 1; Tables S2 and S3). PCA explains 82.39% of the variability, illustrating the discriminating capacity of the e-nose. Although the data analysis shows interesting results, the instrument did not allow the molecules detected by each sensor to be identified.

### 2.4. Brewing Trial Analysis

Before dry hopping, the beer was characterised by the following technological parameters: Original Extract 15.9% (*w*/*w*), Apparent Extract 3.4–3.1% (*w*/*w*), Real Extract 5.5–5.9% (*w*/*w*), Alcohol 6.8–7.0% (*v*/*v*), CO_2_ 4.6–0.8 (g/L), O_2_ 0.181–0.193 (g/L) and Color value 12 (EBC unit).

#### 2.4.1. HS/SPME/GC-MS Analysis of Conditioned Dry-Hopped Beers

Differences in hop aroma profiles were the result of β-myrcene, α-humulene and α-pinene (Table 3), but it is known that the physicochemical properties of these compounds produce a scant contribution to beer aroma [20,32].

With regard to beer, HS-SPME-GC-MS analysis highlighted differences among the studied samples (Figure S1), with only terpenes (3 ± 3.1% of the total peak area) being considered. The compound β-myrcene showed a similar trend in beer (Table 4) when compared to hops (Table 3). Similarly, a higher percentage of β-myrcene in beer was also recorded in BT1 (Figure S1). In contrast, linalool and methyl geranate in beer showed different trends when compared to hop samples, with no significant differences being detected in hops, whereas significant differences were found in beer samples. The aromatic profile of beer is very complex and depends on malt, yeast and hops together with a large number of biotransformation events that take place during the brewing process. In particular, β-myrcene may oxidise and this, through multiple reactions, may generate pinene isomers and terpenoids, such as linalool and geraniol [20]. In Table 4, a significantly higher presence of terpenes in the BC (control beer) can be observed, with the exception of geraniol and methyl geranate, resulting in higher percentages in the beers obtained using hop samples BT1, BT2, BT3 and BT4 (T1 beer; T2 beer; T3 beer; T4 beer). This result shows that, while there was no direct correlation with hop fertilisation treatments regarding the target aromatic compounds, nevertheless, variations were measured in beers that were dry-hopped using different hops.

#### 2.4.2. Cyranose 320 Analysis on Dry-Hopped Beers

**Figure 2:**
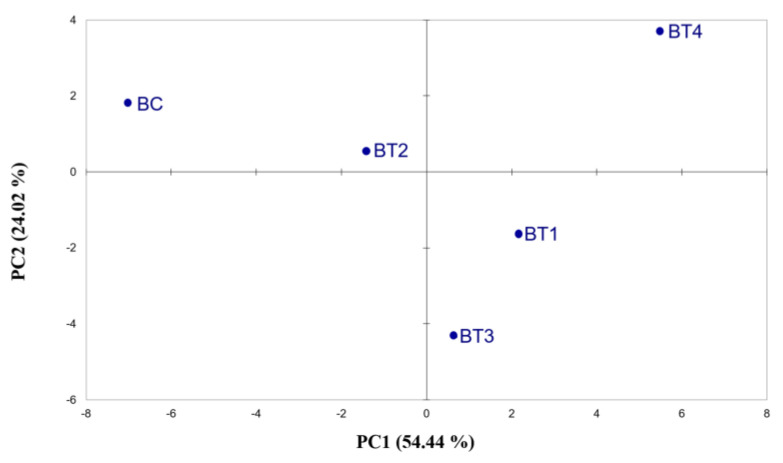
Score plots obtained from the PCA of Cyranose 320 sensor signals on beers made from hops across the fertilisation treatments. The analysis was performed using Pearson’s method (*p* ≤ 0.05). BC = control beer; BT1 = T1 beer; BT2 = T2 beer; BT3 = T3 beer; BT4 = T4 beer.

Similarities were detected between PCA based on Cyranose 320 hops and beer volatile analysis. In beer e-nose analysis (Figure 2 and Tables S2 and S3), the BC sample was shown to be more distant from the others but not as isolated as in the previous analyses (Figure 1). In addition, BT4 was to be found at a greater distance from the other treatments, highlighting a different response to the e-nose. The results obtained with the electronic nose underline the potential usefulness of this instrument to differentiate between beers; the extreme sensitivity of the instrument enables it to detect small differences in the volatile fraction. Our results are in line with previous studies, where the electronic nose was able to differentiate between fruit juices with different alcohol mixtures added [33] and between cold soak wines [34], thus demonstrating its ability to discriminate volatile variations, which is comparable to SPME-GC-MS.

#### 2.4.3. Sensory Analysis

Sensory scores on the appearance, aroma, taste and overall acceptability of the beer samples are reported in Table 5. All beer samples were considered acceptable by consumers with an acceptability score of more than 5 (neither like nor dislike). In terms of overall acceptability, BT1, BT2, BT3 and BT4 beers were similar to the BC. The respondents were asked to rank the five beers in order of preference, with 1 denoting the most preferred and 5 the least preferred. The ranking sums of the beers are reported in Table 5.

In terms of appearance, all beers scored between 5 (neither like nor dislike) and 6 (slightly like). The rating for the odour and flavour of beer sample BT1 and the BC scored significantly differently from BT2 and BT4, showing a perception of differences between the beers by the consumers.

The rating scores for the odour and aroma of BT4 beer were significantly lower than the BC but were not lower than BT1, BT2 and BT3. The most acceptable beer was BT1, followed by the BC. Odour and flavour revealed significant differences (Table 5): lower values indicate a preference between the analysed samples. In a previous study by Gabrielyan et al. [21], where the impact of under-fertilised hops on consumers’ willingness to purchase beers was evaluated, differences were observed with a preference for the beer produced with standard fertilised hops. Their results highlight the ability of consumers to identify differences among beers made with differently fertilised hops. In our study, the preference ranking of the beers was BT1 > BC > BT4 > BT2 ~ BT3, with a slight preference for BT1 and the C. These data are interesting and partially reflected by the analytical data, as can be seen in Table 4; the BC is richer in linalool and citronellol, whereas BT1 is richer in myrcene.

## 3. Materials and Methods

### 3.1. Experimental Design and Hop Cone Sampling

The experiment was carried out during the 2018/2019 season at the “Azienda Agric-ola Ludovico Lucchi”, located in Campogalliano (MO-IT) (44.704140 N, 10.841347 E). Meteorological data are reported in the Supplementary Materials, Figure S2. In the hop field, plants were spaced 1.2 m from each other along the row, with the rows being 3 m apart. Hop plants were grown on a loamy clay soil, with 1,04% of gravel, coarse sand and fine sand (1 and 12.9%), coarse slit and fine slit (15 and 35%), and 36% of clay. The physical and chemical proprieties of the soil are reported in Table 6. In winter, 30 t·ha^−1^ of cow manure was supplied to the soil. The experimental design and the first results of the experiment regarding physiological measurements have already been published [22]. During the growing season, four organic fertiliser mixtures, based principally on marine algae, amino acids and microelements (T1, T2, T3 and T4) (Table S1), which had been kindly provided by Caprara S.r.l., were tested on the same cultivar (Cascade). Each treatment, consisting of different nutrient mixtures (Table 1, Table S1), was applied utilising an atomiser machine (foliar treatments). The experiment involved two periods where foliar treatment was carried out: the first period (t1) took place between the emission of lateral shoots and the start of blooming; the second period (t2) took place between 50% blooming and the end of blooming. For each period, three foliar treatments, one per week, were applied. The amounts of macro- and microelements applied to the hop plants are indicated in Table 1. Non-treated plants were also considered, representing the control. For each treatment, 3 independent replicates were used, with each replicate consisting of 150 plants. Each treatment was separated from the next by 5 rows of untreated plants.

### 3.2. Hop Cone Sampling

The hop cones were harvested and divided per treatment (13 September 2019). They were then immediately dried by the farmer at 50 °C for 8 h. In order to ensure the robustness of the data, the hop cones for each replicate were derived from 150 plants. Three replicates for each treatment were considered. After drying, 1 kg of homogenised sample from each replicate was stored in vacuum-sealed packages at −20 °C.

### 3.3. Hop Cone Analysis

#### 3.3.1. Hop Oil GC-MS Analysis

Essential oils were first extracted in triplicate by steam distillation with a Clevenger apparatus, using 25.0 g of dried hops left for 4 h in a 2.0 L round-bottom flask with 1250 mL of pure water. Anhydrous sodium sulfate (Sigma-Aldrich, Milan, Italy) was then added to the extracted essential oils, which were subsequently stored at 4 °C. Before analysis, the essential oils were diluted in CH_2_Cl_2_ (1:200 *v*/*v*), to which 400 ppm of Toluene had been added as the internal standard (Carlo Erba, Milan, Italy).

All samples were analysed with a Thermo Scientific (San Jose, CA, USA) TRACE 1300 gas-chromatograph coupled to a Thermo Scientific ISQ™ Single Quadrupole mass spectrometer. The gas-chromatograph was equipped with Supelcowax 10 (30 m × 0.25 mm, f.t. 0.25 μm) (Supelco, Bellefonte, PA, USA) capillary columns and helium was used as a carrier gas (1 mL min^−1^). The gas chromatography–mass spectrometry (GC–MS) oven temperature gradient started from 50 °C, a condition that was maintained for 3 min, after which the temperature was raised to 200 °C (5 °C min^−1^). This final temperature was maintained for 18 min. The injector was maintained at 230 °C, operating in split modality with a ratio of 1:20. The mass spectrometer was equipped with an electron impact (EI) source (70 eV) and the acquisition mode was full scan (from 40 to 500 *m*/*z*). A solvent delay time of 4 min was applied. The main terpenic volatile compounds were identified on the basis of their mass spectra compared with the reference mass spectra libraries (NIST, 2005 software, Mass Spectral Search Program V.2.0d, Washington, DC, USA version 2.2 June 2014) and on the basis of their calculated Retention Indexes (RIs) through the application of the Kovats’ index (KI) formula compared with those reported in the literature [25,35,36]. When it was not possible to find the KI in the literature, a tentative identification was obtained by matching the value obtained with mass spectra library data: a minimum match quality of 98% was used as a criterion. In order to determine the RI of the components, a mixture of alkanes (C8–C20) was injected in the GC–MS equipment and analysed under the same conditions described earlier. After the identification of target volatile compounds (VOC), peak areas of target volatile compounds (AVOCs) were obtained on the basis of the major ion then normalised with the internal standard peak area (AIS). Data were expressed as a percentage based on the equation below:

(A_voc_/A_SI_/∑ A_voc_/A_SI_) ∗ 100

A_voc_ = integrated peak area

A_SI_ = area of the internal standard

All samples were analysed in triplicate.

#### 3.3.2. Hop Cone Electronic Nose Analysis

The electronic nose employed for the analysis was the Cyranose 320 (Sensigent LLC, 1438 Arrow Hwy, Baldwin Park, CA, USA, 91706). The Cyranose 320 integrates 32 polymer sensors, which, when in contact with a vapour, absorb the vapour and swell like a sponge. During this process, the electronic nose arrays are used to identify the vapour [37]. The ability of the electronic nose to discriminate different samples has been previously observed, for example, in long-grain rice, where it was able to discriminate the cultivars [37]. In another study, the electronic nose was successfully used with apples to distinguish fruit ripeness grades [38,39]. In our study, the electronic nose was used to analyse and characterise the samples. Cyranose 320 software was used to record the experimental data. Six dried cones per sample in triplicate were placed into a 50 mL falcon, which was then closed with the internal headspace equilibrated for 30 min. Measurements could be registered when the resistance of the gas sensors remained stable at a high value of 0~1 [37].

Before modelling, the cross-validation technique was applied to determine the optimum number of principal components. The results were used in principal component analysis in order to classify the samples and identify differences.

### 3.4. Brewing Trials

Dry hopping, which consists of adding hops to the cold liquor at the end of the main fermentation process, was used to emphasise and preserve the hop aroma [40]. This is a relatively easy method, used to enhance the flavour and fragrance of beer. The chemical procedure improves both the aroma and the microbial stability of beer by the cold extraction of volatile and non-volatile compounds [41].

The control hops, together with the hops coming from the four fertilisation treatments, were used and dry-hopped to obtain five different beers (BC, BT1, BT2, BT3 and BT4).

#### Brewing Process

Two batches of India Pale Ale beer, each of 100 L, were produced at the pilot plant facility of Porto Conte Ricerche Srl (Alghero, Italy). Grist containing 90% Pale Ale malt (Weyermaan, Bamberg, Germany), 8% Carared malt (Weyermaan, Bamberg, Germany) and 2% Carapils malt (Weyermaan, Bamberg, Germany) was ground in a two-roll mill with 1 mm spacing. Mashing was carried out using 75 L of water to which 20 g of CaSO_4_ (Mr. Malt, Udine, Italy) and 10 g of CaCl_2_ (Mr. Malt, Udine, Italy) were added. Mashing was conducted at 66 °C for 60 min, heated to a temperature of 78 °C, and then kept for 10 min for mash-out. The wort was first transferred to a kettle, and then spent grain was washed using hot water at 78 °C. The Cascade hop (Mr. Malt, Udine, Italy) was added at the start of boiling for bittering at the final international Bitter Unit (IBU) of 30. The boiled wort was separated from the hot trub by whirlpool then cooled at 18 °C.

Wort fermentation was conducted in different vessels with 20 L of boiled wort for each experimental treatment. Dry yeast Saf-Brew US-05 (Fermentis, Marcq-en-Baroeul Cedex, France) was added directly to the fermenter (0.5 g L^−1^), after which fermentation was carried out at 15 °C for seven days to obtain green beer. The temperature was then reduced to 4 °C for two weeks to obtain cold-matured beers.

Tanks of 20 L of green beer were dry-hopped for 5 days at 4 °C using 300 g of each hop sample (C, T1, T2, T3 and T4) in duplicate. After this, the hops were removed and the beers were bottled. Conditioned dry-hopped beer was obtained by adding 6 g L^−1^ of glucose and 0.05 g L^−1^ of yeast F2 (Fermentis, Marcq-en-Baroeul Cedex, France) and keeping the beer at 22 °C for 14 days.

### 3.5. Single-Hopped Beer Analysis

#### 3.5.1. Determination of Standard Quality Attributes

The original extract (% *w*/*w*), apparent extract (% *w*/*w*), real extract (% *w*/*w*) and alcohol (% *v*/*v*) were measured in triplicate with a PBA-B generation M (Anton Paar, Graz, Austria). The following analyses were performed according to the official Analytica-European Brewery Convention methods [42]: colour (EBC-U) using EBC method 9.6; pH using EBC method 9.35; and turbidity (EBC-U) using EBC method 9.29. In addition, foam stability was measured with a NIBEM-OPH foam stability tester (Haffmans, Zeist, The Netherlands) according to Analytica-EBC method 9.42.1.

All analyses were performed in triplicate.

#### 3.5.2. HS/SPME/GC-MS Analysis

The five beers (BC, BT1, BT2, BT3 and BT4) were analysed for their volatile compounds in triplicate. Five mL of beer was transferred into 10 mL headspace vials containing 1.5 g of sodium chloride and 10 µL of 1-Butanol as the internal standard (25 g/L), which were then sealed with PTFE–silicone septa and stored at 7 °C in a refrigerated compartment. Analysis of volatile compounds was carried out using the headspace solid-phase microextraction (HS/SPME/GC/MS) technique by means of Divinylbenzene/Carboxen/Polydimethylsiloxane (DVB-CAR-PDMS) fibre (Supelco, Bellefonte, PA, USA) [43]. For SPME analysis, the samples were incubated for 10 min at 40 °C, after which extraction was carried out exposing the fibre to the headspace for 40 min. Both incubation and extraction were performed by agitation. Fibre desorption was carried out in an injector for 2 min at 250 °C with a split flow of 7.2 mL min^−1^. The fibre was activated each day following the manufacturer’s instructions. Chromatographic analysis was performed using TRACE GC coupled with an ISQ single quadrupole (Thermo Scientific, Hudson, NY, USA). The analytes were separated on a SLB-5 ms capillary column (60 m × 0.25 mm × 0.25 μm film thickness) (Supelco, Bellefonte, PA, USA) using helium as a carrier gas at a 1.2 mL min^−1^ constant flow rate. The oven temperature programme started at 50 °C and was held there for 5 min and then it was increased at 5 °C min^−1^ to 250 °C and held there for 3 min [44]. The transfer line and ion source were set at 250 °C and 270 °C, respectively, with a quadrupole scan ranging between 33 and 300 amu and with the ionisation energy being 70 eV [45]. Chromatographic data were acquired by means of Tracefinder (Thermo Scientific, Waltham, MA, USA).

The identification of terpenes was carried out by comparing the mass spectra with those of the data system library (NIST, 2005 software, Mass Spectral Search Program V.2.0d, Washington, DC, USA version 2.2 June 2014) [36,46]. All Identity Spectrum Match factors above 850 resulting from the NIST Identity Spectrum Search algorithm (NIST MS Search 2.0) were considered acceptable for positive identification. After the identification of target volatile compounds (VOCs), peak areas (A_VOC_) were obtained on the basis of the major ion then normalised with the internal standard peak area (A_IS_). Data were expressed as a percentage on the basis of the Equation explained in Section 3.3.1.

#### 3.5.3. Beer Electronic Nose Analysis

For instrument training, 15 mL of beer per sample in triplicate was placed in a 50 mL falcon. The falcon was then closed and the headspace inside was equilibrated for 45 min. The analysis was performed as reported in Section 3.3.2.

#### 3.5.4. Sensory Analysis

Two methods of hedonic evaluation were employed: Method I involved a nine-point hedonic scale, anchored at both extremes where 1 = “extremely dislike” and 9 = “like very much”; Method II involved a classification of samples according to their degree of approval.

A hedonic survey of the consumer acceptability of the five beers was conducted in the Food Science Laboratory of Porto Conte Ricerche Srl (Alghero, Italy). The sensory scores of the four beer samples (BT1, BT2, BT3 and BT4) and the control BC were evaluated using an acceptance test and a nine-point hedonic scale. The consumers gave a score of 1–9 to the samples, ranging from “extremely dislike” to “like very much” [47]. A total of 52 subjects were included in the consumer study, 36 males and 16 females aged 32–60 years. They were asked to fill out an anonymous questionnaire with their demographic data (age, gender, education level, specific food frequency questions). A questionnaire was also provided to obtain their opinion of the beer samples, preferences and purchase intent. The consumers recruited for the test declared that they habitually consumed craft beers and that they paid particular attention to the olfactory and gustatory characteristics of beers [48].

Before participating in the study, all the consumers provided their informed consent. The prepared samples were stored at 4 ± 2 °C until evaluation; the samples were presented in glass beakers equipped with lids to limit the loss of volatile compounds and coded with three-digit random numbers in a randomised presentation order to minimise bias. The respondents were then asked how much they liked the beers in terms of appearance, odour, flavour and overall acceptability.

Before each evaluation, the subjects were briefed on each of the two methods for the task and on the tasting technique (taste and swallow). Mineral water at room temperature was available as a neutraliser between samples. In Method I, the subjects rated the agreeableness (degree of liking) of each sample on the nine-point hedonic scale. The consumers were instructed to take only as much of each sample as needed to make an assessment during the test. In Method II, after tasting all five samples, the consumers were asked to rank the beers in order, from the most-liked to the least-liked sample. The subjects were then instructed to taste the beer samples by taking a sip of each, after which they had to attempt to grade the samples based on their first sensory impressions, repeating the test (if necessary) to ensure correct grading.

Classification data were analysed with Friedman’s test of rank to find out if there were significant differences in agreeableness between samples and to determine which samples differed from each other (ISO8587, 2006).

### 3.6. Statistical Analysis

All data obtained were evaluated using XLSTAT (Addinsoft SARL, Long Island City, NY, USA) and Statgraphics Centurion XV (Manugistics Inc., Rockville, MD, USA). The results were statistically analysed using the Kruskal–Wallies and Dunn’s post hoc tests according to variance homogeneity (Shapiro–Wilk test and Cochran test), with a cut-off significance of *p* < 0.05. For the aromatic profiles of both hops and beer (data of GC and Cyranose 320), a Kaiser–Meyer–Olkin test (KMO) and Barlett’s test were first employed, followed by principal component analysis (PCA) using Pearson’s method (*p* ≤ 0.05). Classification data on sensory analysis was performed with Friedman’s test of rank.

## 4. Conclusions

This study illustrates the effects of four fertilisation treatments and a control on the aromatic profile of Cascade hops and, consequently, on the aroma and flavour of five single dry-hopped beers. Modifications could be observed regarding the presence of a number of terpenes in the volatile fraction in both hops and beers, using not only standard analytical methods (GC-MS and HS-SPME-GC-MS) but also an electronic portable nose (Cyranose 320). The results showed that the variations between hops and beer samples, even if small, are detectable using different methods. Terpene analyses on hop cones revealed a major presence of monoterpenes and sesquiterpenes in the control and in the fertilised hops, respectively. Sensory analysis carried out on consumers determined the acceptability of the produced beers, showing that differences among the samples were perceptible by consumers themselves. The scores revealed that significant differences were perceived in both odour and flavour, thus emphasising the high impact that the nutritional treatments applied to hops have on the beers produced. In the sensory analysis, preference was given to the BT1 sample, while the instrumental analysis showed a higher presence of volatiles in the control, with the exception of geraniol. Nonetheless, in the sensory evaluation, all the tested samples were evaluated as acceptable.

## Figures and Tables

**Table 1:** Macro- and microelements (g ha^−1^) included in every nutrient mixture per treatment [22].

Treatment	N Org.	P_2_O_5_	K_2_O	Mg Chel	Zn Solub	Zn Chel	Mn Solub	Mn Chel	Fe Solub	Fe Chel.	Cu Solub	Cu Chel	B Solub	Mo Solub
T1	30	1125	2070	360	45	45	120	120	120	120	45	45	15	18
T2	16,230	-	570	270	45	45	120	120	120	120	45	45	15	3
T3	16,080	3600	19,170	180	21	21	-	-	1950	1950	-	-	-	-
T4	16,080	-	16,770	-	66	66	120	120	2070	2070	45	45	345	3

C = control treatment; T1 = Treatment 1; T2 = Treatment 2; T3 = Treatment 3; T4 = Treatment 4.

**Table 2:** Bitter acid (α- and β-acids) content (% *w*/*w* per dry weight), cohumulone (COH, % on the total of alpha acids) and essential oil yield (% *v*/*w*) of the studied hop samples [22] and moisture content (% *w*/*w*), means and standard deviation.

Treatment	α-Acids %	β-Acids %	COH %	Ess. Oil Yield %	Moisture %
C	4.3 ± 0.3 c	5.3 ± 0.2 b	26.2 ± 0.5 a	1.1 ± 0.1 c	11.1 ± 0.1 a
T1	5.8 ± 0.4 b	4.9 ± 0.5 b	26.7 ± 0.5 a	1.4 ± 0.1 b	10.0 ± 0.1 a
T2	7.0 ± 0.3 a	6.4 ± 0.1 a	26.8 ± 0.1 a	1.8 ± 0.1 a	10.1 ± 0.2 a
T3	5.8 ± 0.6 b	5.3 ± 0.2 b	26.1 ± 0.4 a	1.5 ± 0.2 b	10.5 ± 0.2 a
T4	5.1 ± 0.1 bc	5.05 ± 0.2 b	26.2 ± 0.4 a	1.3 ± 0.1 b	10.1 ± 0.1 a

C = control treatment; T1 = Treatment 1; T2 = Treatment 2; T3 = Treatment 3; T4 = Treatment 4. Different letters indicate significant differences with a cut-off significance of *p* < 0.05.

**Table 3:** Relative % abundance of hop sample terpenes. Means ± standard deviation of the percentage of total sum area of the integrated terpenes peak area (A_S_) compared to the area of the internal standard (A_IS_). Different letters indicate significant differences using Kruskal–Wallis and Dunn’s post hoc tests, with a cut-off significance of *p* < 0.05.

Terpenes	C	T1	T2	T3	T4	RI Calc *
α-Pinene	0.1 ± 0.03 a	0.2 ± 0.01 a	0.3 ± 0.00 a	0.2 ± 0.05 a	0.3 ± 0.01 a	1028
β-Pinene	1.3 ± 0.22 b	1.8 ± 0.02 ab	1.9 ± 0.02 ab	1.8 ± 0.20 ab	2.0 ± 0.00 a	1039
β-Myrcene	47.3 ± 3.01 b	58.5 ± 1.73 a	57.9 ± 1.36 ab	57.4 ± 1.39 ab	56.5 ± 1.10 ab	1173
Limonene	1.0 ± 0.07 a	1.2 ± 0.05 a	1.1 ± 0.04 a	12 ± 0.06 a	1.2 ± 0.06 a	1185
β-Linalool	0.7 ± 0.19 a	0.7 ± 0.03 a	0.8 ± 0.03 a	0.7 ± 0.03 a	0.7 ± 0.01 a	1544
β-Caryophyllene	6.5 ± 1.08 a	4.8 ± 0.60 ab	4.3 ± 0.04 b	4.6 ± 0.24 ab	5.2 ± 0.60 ab	1577
trans-Bergamotene	0.7 ± 0.14 a	0.4 ± 0.07 a	0.5 ± 0.04 a	0.5 ± 0.04 a	0.5 ± 0.07 a	1581
trans- β- Farnesene	6.3 ± 0.60 a	4.6 ± 0.63 a	4.6 ± 0.41 a	4.7 ± 0.14 a	4.9 ± 0.23 a	1645
α-Humulene	15.2 ± 2.25 a	9.6 ± 0.96 ab	9.0 ± 0.57 b	10.0 ± 1.08 ab	11.6 ± 2.06 ab	1658
Muurolene	1.1 ± 0.13 a	1.1 ± 0.29 a	0.8 ± 0.04 b	0.7 ± 0.00 b	0.8 ± 0.04 ab	1684
Methyl geranate	0.8 ± 0.20 a	0.7 ± 0.01 a	0.7 ± 0.08 a	0.7 ± 0.00 a	0.7 ± 0.06 a	1688
β-Selinene	2.1 ± 0.21 a	1.3 ± 0.79 ab	1.8 ± 0.03 ab	1.7 ± 0.08 ab	1.7 ± 0.06 b	1714
α-Selinene	3.4 ± 0.11 a	2.6 ± 1.13 ab	2.9 ± 0.01 ab	2.7 ± 0.04 b	2.9 ± 0.05 ab	1718
τ-Cadinene	0.9 ± 0.11 a	0.9 ± 0.61 ab	0.6 ± 0.04 b	0.6 ± 0.03 b	0.7 ± 0.02 ab	1755
δ-Cadinene	1.5 ± 0.22 a	0.9 ± 0.38 a	0.9 ± 0.08 a	0.9 ± 0.01 a	1.0 ± 0.07 a	1763
trans-1,4-Cadina diene	0.1 ± 0.03 a	0.5 ± 0.62 a	0.1 ± 0.01 a	0.1 ± 0.00 a	0.1 ± 0.03 a	1769

* RI calc = calculated retention index, in accordance with the literature [24,25]. C = control treatment; T1 = Treatment 1; T2 = Treatment 2; T3 = Treatment 3; T4 = Treatment 4.

**Table 4:** Relative % abundance of terpenes of beer samples. Means ± standard deviation of the percentage of total sum area of the integrated peak area (AS) compared to the area of the internal standard (AIS). Different letters mean significant differences using Kruskal–Wallis and Dunn’s post hoc tests, with a cut-off significance of *p* < 0.05.

Terpenes	BC	BT1	BT2	BT3	BT4	RI Calc *
β-Myrcene	0.4 ± 0.19 a	0.6 ± 0.09 a	0.3 ± 0.08 a	0.3 ± 0.12 a	0.2 ± 0.03 a	991
Linalool	1.3 ± 0.15 a	0.9 ± 0.06 bc	0.9 ± 0.00 c	0.9 ± 0.01 abc	1.1 ± 0.01 ab	1099
Terpineol	0.2 ± 0.05 a	0.1 ± 0.00 b	0.1 ± 0.00 b	0.1 ± 0.00 b	0.1 ± 0.00 b	1188
Citronellol	1.1 ± 0.08 a	07 ± 0.07 ab	0.6 ± 0.04 b	0.7 ± 0.02 ab	0.6 ± 0.02 ab	1228
Geraniol	0.2 ± 0.02 b	0.3 ± 0.04 a,b	0.4 ± 0.05 a	0.4 ± 0.05 a	0.4 ± 0.01 a	1255
Methyl geranate	0.1 ± 0.02 b	0.4 ± 0.01 a	0.3 ± 0.01 ab	0.4 ± 0.00 a	0.4 ± 0.01 a	1302

* RI calc = calculated retention index, in accordance with the literature [24,25]. BC = control beer; BT1 = T1 beer; BT2 = T2 beer; BT3 = T3 beer; BT4 = T4 beer.

**Table 5:** Mean hedonic scores of sensory attributes of appearance, aroma, flavour and overall acceptability are tabulated. Values are arithmetic means ± standard deviation (N = 52 consumers, who evaluated beers in a structured nine-point hedonic scale). Different letters indicate significant (*p* < 0.05) differences according to Fisher’s LSD test.

Hedonic Score for Beer Samples					
Sensory Attribute	BC	BT1	BT2	BT3	BT4
Appearance	5.6 ± 1.5 a	5.8 ± 1.3 a	5.7 ± 1.2 a	5.7 ± 1.1 a	5.8 ± 1.3 a
Odour	6.1 ± 1.2 b	6.0 ± 1.3 b	5.1 ± 1.3 ab	5.4 ± 1.3 ab	5.2 ± 1.0 a
Flavour/aroma	5.7 ± 1.3 b	5.8 ± 1.3 b	5.1 ± 1.2 a	5.3 ± 1.3 ab	5.1 ± 1.3 a
Taste	5.3 ± 1.3 a	5.4 ± 1.3 a	5.3 ± 1.3 a	5.2 ± 1.3 a	5.4 ± 1.3 a
Overall Acceptability	6.0 ± 1.2 a	6.1 ± 0.9 a	5.7 ± 1.1 a	5.8 ± 1.4 a	5.8 ± 1.3 a
**Preference +**	166	164	174	174	172

+ Lower ranking denotes greater preference by respondents.

**Table 6:** Chemical properties of the soil and elements.

**Parameter**	**Value**	**Unit**
pH	7.33	
EC	264	µS/cm
CEC	30.86	cmol_c_/Kg
**Element**	**Value**	**Unit**
(N) tot	2	g/Kg
Organic matter	3.1	% *w*/*w*
(P) available	17.8	mg/kg
(P_2_O_5_) available	40.9	mg/kg
Ca	13.65	mmol/L
Mg	1.1	mmol/L
K	1.23	mmol/L
Na	0.22	mmol/L
Zn	41	mg/kg
Fe	17.4	g/kg
B	11	mg/kg

EC = Electric Conductivity; CEC = Cation Exchange Capacity.

## Data Availability

Not applicable.

## Supplementary Materials

The following supporting information can be downloaded at: https://www.mdpi.com/article/10.3390/plants12091861/s1, Figure S1: GC-MS, HS-SPME-GC-MS outputs; Figure S2: Meteorological data; Table S1: Fertilizers characteristics and compositions; Table S2: PCAs variability; Table S3: e-nose output.
