# Supplementary material for: From Hop to Beer: Influence of Different Organic Foliar Fertilisation Treatments on Hop Oil Profile and Derived Beers’ Flavour
Source: Plants (Basel). 2023 Apr 30;12(9):1861. doi: 10.3390/plants12091861 (PMC10180877; doi:10.3390/plants12091861)

Supplementary Material S1

Figure S1. Overlaid chromatograms of the GC-MS analysis on hop essential oils with Internal Standard (I.S.) highlighted. Each different line color correspond to a different treatment.

Legend:

CF3 (black line)= Control

T1F2 (red line)= T1 treatment

T2F1 (green line)= T2 treatment

T3F3 (blue line)= T3 treatment

T4F1 (ocher line)= T4 treatment

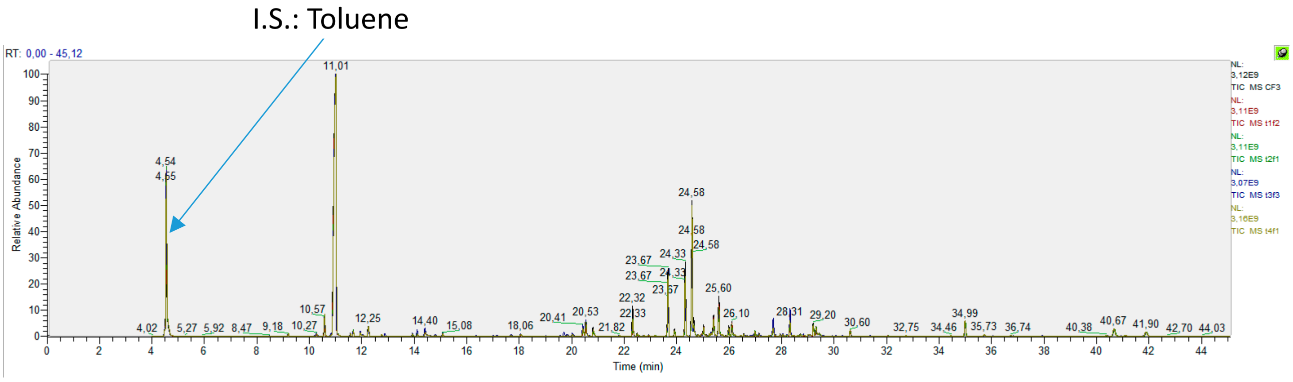

Figure S2. Overlaid chromatograms of the HS-SPME-GC-MS analysis on beers with Internal Standard (I.S.) highlighted. Each different line color correspond to a different treatment.

Legend:

BC (green line)= Beer with hop from control

BT1 (black line)= Beer with hop from treatment 1

BT2 (blue line)= Beer with hop from treatment 2

BT3 (red line)= Beer with hop from treatment 3

BT4 (ocher line)=Beer with hop from treatment 4

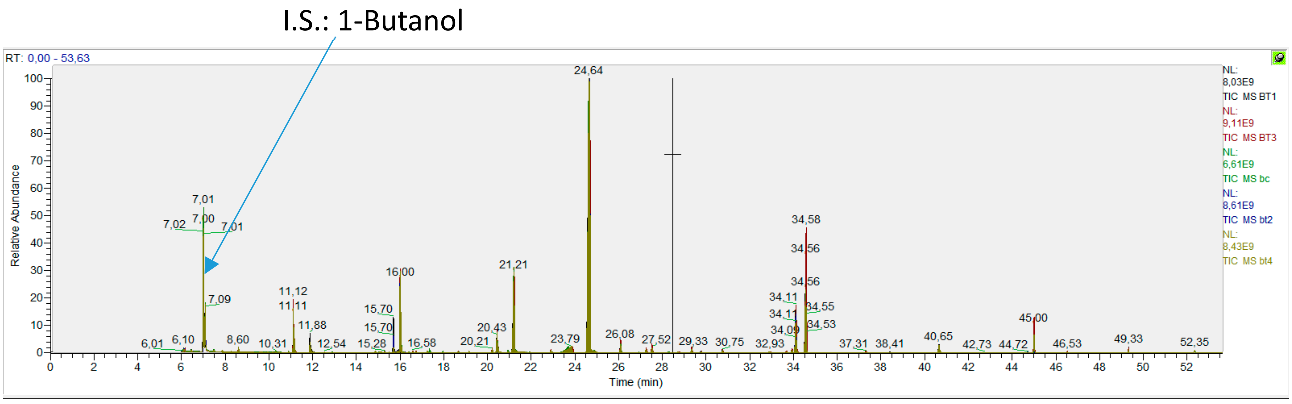

Figure S3. Chromatograms detail of the differences in Myrcene content in beer samples. The highest peak is owned by BT3 (in black), the lowest peak correspond to BT4 (in ocher).

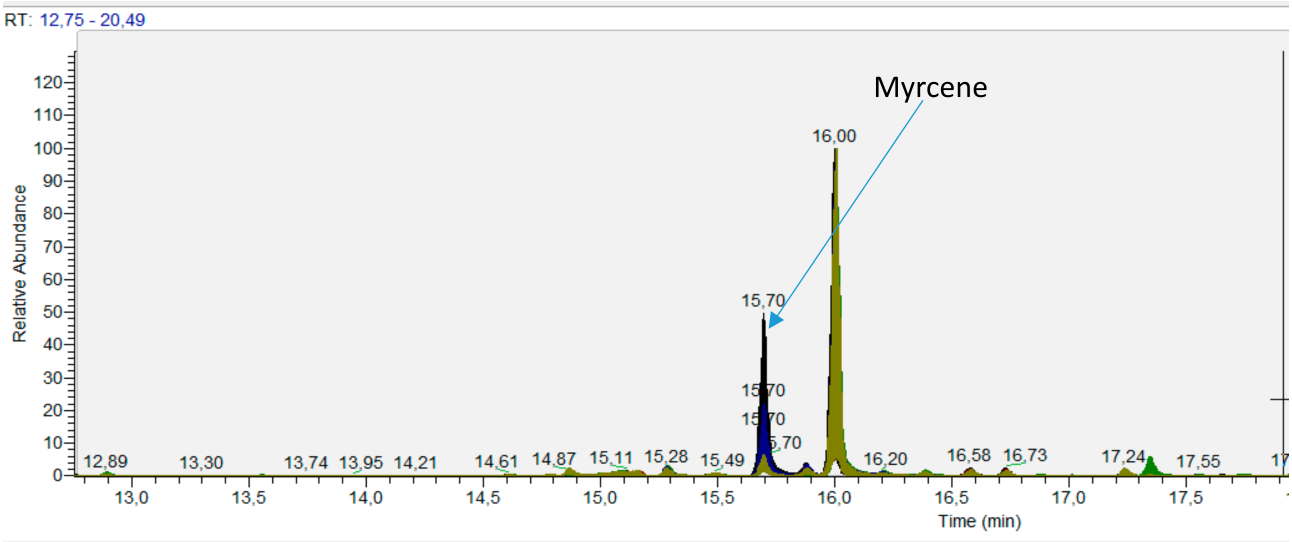

Supplement: Supplementary file 1 [file plants-12-01861-s001.zip › Figure S1_chromatograms.pdf]
