# Supplementary figures and images for: From Hop to Beer: Influence of Different Organic Foliar Fertilisation Treatments on Hop Oil Profile and Derived Beers’ Flavour
Source: Plants (Basel). 2023 Apr 30;12(9):1861. doi: 10.3390/plants12091861 (PMC10180877; doi:10.3390/plants12091861)

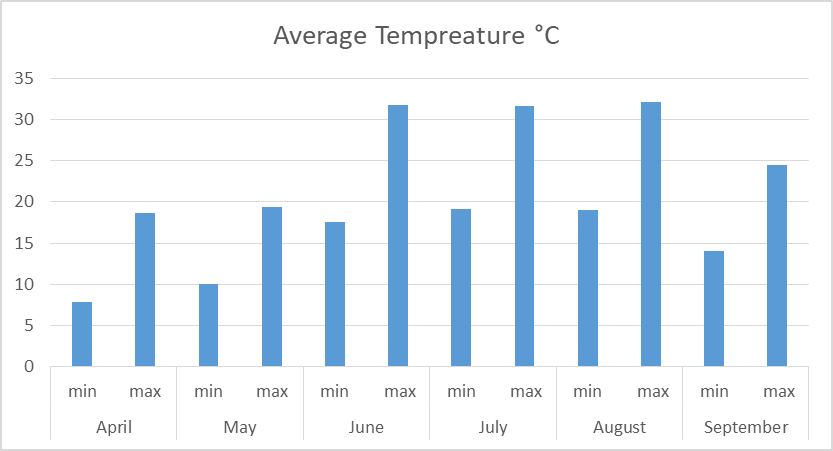

Supplement: Supplementary file 1 [file plants-12-01861-s001.zip › Figure S2_Meteorological data.png]
